# Co‐Expression of Tardive Dyskinesia and Drug‐Induced Parkinsonism in Rats Chronically Treated With Haloperidol

**DOI:** 10.1002/npr2.12524

**Published:** 2025-01-09

**Authors:** Iku Kinoshita, Haruo Nishijima, Takashi Nakamura, Tomoya Kon, Shuji Shimoyama, Hiroki Hikichi, Chieko Suzuki, Masahiko Tomiyama

**Affiliations:** ^1^ Department of Neurology Hirosaki University Graduate School of Medicine Hirosaki Japan; ^2^ Department of Neurology Hirosaki University Hospital Hirosaki Japan; ^3^ Department of Neurophysiology Hirosaki University Graduate School of Medicine Hirosaki Japan

**Keywords:** direct pathway, dopamine D1 receptor, dopamine D2 receptor, dynorphin, enkephalin, indirect pathway, vacuous chewing movement

## Abstract

**Aim:**

We aimed to create a rat model of drug‐induced parkinsonism and tardive dyskinesia by chronic administration of haloperidol and examine the expression of direct and indirect pathway markers in the striatum of the model rats.

**Methods:**

We treated 21 rats, 14 with haloperidol decanoate and 7 with placebo. The number of vacuous chewing movements per 2 min was counted, and haloperidol‐treated rats were classified into two groups: mild and severe tardive dyskinesia. Other behavioral analyses were also conducted. After a 6‐month treatment period, rat brains were removed, and protein expression was evaluated by Western blotting.

**Results:**

All haloperidol‐treated rats exhibited vacuous chewing movements. The frequency of exploratory behavior and rotarod test performance was lower in the mild and severe tardive dyskinesia groups. The number of vacuous chewing movements and frequency of exploratory behavior were positively correlated in haloperidol‐treated rats. The expression of dynorphin, a direct pathway marker, decreased in the severe tardive dyskinesia group. The expression of enkephalin, an indirect pathway marker, decreased both in the mild and severe tardive dyskinesia groups. The expression of dopamine D1 and D2 receptors also decreased with haloperidol treatment.

**Conclusion:**

Both direct and indirect pathways are involved in haloperidol‐induced movement disorders.

## Abstract 2

Rats chronically treated with haloperidol exhibit drug‐induced parkinsonism and tardive dyskinesia. Expression of dynorphin, enkephalin, and dopamine D1 and D2 receptors was altered in haloperidol‐treated rats. Both indirect and direct pathways are involved in haloperidol‐induced movement disorders.

## Graphical abstract

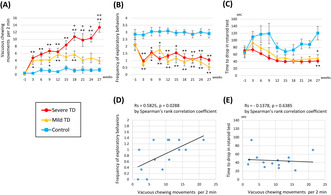

**Abbreviations**
ANOVA: analysis of variance
DIP: drug‐induced parkinsonism
FGA: first‐generation antipsychotic
GABA: gamma‐aminobutyric acid
GAPDH: glyceraldehyde 3‐phosphate dehydrogenase
RNA: ribonucleic acid
SGA: second‐generation antipsychotic
TD: tardive dyskinesia
TH: tyrosine hydroxylase
VCM: vacuous chewing movement

## 1 Introduction

Recently, prescriptions for antipsychotic drugs, especially second‐generation antipsychotics (SGAs), have been increasing [1]. Although SGAs may have fewer side effects than first‐generation antipsychotics (FGAs), they cause drug‐induced parkinsonism (DIP) and tardive dyskinesia (TD). DIP occurs in 50%–75% of patients taking FGAs within 1 month of initiation and 90% within 3 months [2], whereas it occurs in 20%–35% of patients on SGAs [3]. TD prevalence in patients using antipsychotics is 30% with FGA and 20% with SGA [4]. Nearly 27% of patients with TD have concomitant DIP [5]. DIP is caused by dopamine‐depleting drugs or dopamine receptor blockers such as haloperidol, with symptoms characterized by slow movement and muscle rigidity, which usually disappear within 6 months of discontinuation of the causative drugs [6]. TD is a repetitive involuntary movement that appears in patients using antipsychotic drugs for a long time or at high doses, and may persist after their discontinuation [3].

The pathomechanism of haloperidol‐induced movement disorders is thought to be the blockade of D2 receptors, leading to decreased movement, DIP, which in turn leads to compensatory D2 receptor hypersensitivity, resulting in excessive movement, TD. Thus, changes in the indirect pathway functioning via altered D2 receptor activity in the basal ganglia circuit are considered to cause DIP and TD [7, 8, 9]. In contrast, Gunne et al. [5] proposed that malfunction of the direct pathway is also involved in the pathomechanism of TD; however, there are few studies that prove the involvement of the direct pathway [5, 10].

We aimed to establish a rat model of haloperidol‐induced DIP and TD and examined the association between DIP and TD. We also evaluated the expression of dynorphin and dopamine D1 receptor, direct pathway markers [11], and enkephalin and dopamine D2 receptor, indirect pathway markers [11], in the striata of the rat model.

## 2 Methods

### 2.1 Animals

Twenty‐one male Wistar rats (CLEA Japan Inc., Tokyo, Japan) were used in this study. All efforts were made to minimize the number of animals used and their suffering.

### 2.2 Drug Treatment and Behavioral Analysis

Fourteen rats received haloperidol decanoate (Toronto Research Chemicals Inc., H103710, Toronto, Canada), and seven rats received placebo. Haloperidol, dissolved in sesame oil (FUJIFILM Wako Pure Chemical Corporation, Osaka, Japan) at 100 mg/mL, was administered at a dose of 28.5 mg/kg (0.285 mL/kg solution) via intramuscular injection. Placebo was administered at 0.285 mL/kg with sesame oil. The dose of haloperidol was determined according to previous studies that induced TD‐like behavior in rats [12, 13]. Dosing was initiated at an age of 7 weeks and administered once every 3 weeks for 27 weeks (nine doses in total). Animals were sacrificed 3 weeks after the final dose.

After haloperidol administration, rats gradually developed empty vacuous chewing movements (VCMs), which are involuntary movements causing the mouth to squirm. We considered this movement as TD, as per a previous report [14]. The frequency of this movement was counted from the age of 7 weeks (preceding first haloperidol administration) until sacrificing, once weekly (28 times in total). Animals were videotaped in a 25 × 25 × 25‐cm transparent box and observed for 2 min after a 5‐min acclimation period. Values obtained once before the first haloperidol administration and the average of three measurements after each haloperidol administration were included in the analysis. The average of the three sessions from the ninth dose of haloperidol was used to divide the haloperidol‐treated rats into two groups: severe TD with eight or more VCMs per 2 min and mild TD with fewer than eight VCMs per 2 min.

We evaluated exploratory behavior by observing the animals in the same movies that were shot for evaluating VCMs. The frequency of exploratory behavior was evaluated on a 5‐point scale (constantly: 4; often [> 50% of observation period]: 3; occasionally [< 50% of observation period]: 2; slightly [1–2 small movements]: 1; none: 0).

In the rotarod test using an MK‐630B single‐lane rotor rod (Muromachi Machinery Co., Tokyo, Japan), the time to drop was measured with a rotation speed that started at 4 rpm and increased to 40 rpm after 5 min. Rotarod test trials were performed six times at 20–30‐min intervals. The highest scores among the six trials were analyzed. Rotarod tests were performed once a week, a total of 28 times, starting at 7 weeks of age. The values obtained once before the first haloperidol administration and the average of three values after each haloperidol administration were used for analysis.

### 2.3 Western Blotting

Western blotting was performed for samples from five animals in the control group and six animals each in the mild and severe TD groups. Three weeks after the last drug administration, the rats were sacrificed by overdosing on pentobarbital sodium salt (NACALAI TESQUE, Kyoto, Japan). After intracardial perfusion with saline, rat brains were removed.

Tissues from the left striatum were removed, immediately frozen on powdered dry ice, and stored at −80°C. Protein expression was evaluated by Western blotting, which was performed as described previously [15]. Rabbit polyclonal antibody to dynorphin A (ab82509; Abcam, Cambridge, England; 1:5000) and rabbit polyclonal anti‐enkephalin/ENK antibody (ab85798; Abcam; 1:5000) were used. Additionally, we used anti‐dopamine D1 receptor antibody (17934‐1‐AP; Proteintech Group Inc., Rosemont, IL, USA; 1:5000) and anti‐dopamine D2 receptor antibody (55084‐1‐AP; Proteintech Group Inc.; 1:5000) to evaluate direct and indirect pathway dysfunction. We also used an anti‐tyrosine hydroxylase (TH) antibody (#58844; Cell Signaling Technology, Danvers, MA, USA; 1:5000) as a marker of dopaminergic nerves. Glyceraldehyde 3‐phosphate dehydrogenase (GAPDH; GA1R; Thermo Fisher Scientific, Boston, MA, USA; 1:10000) was used as the housekeeping gene.

### 2.4 Statistical Analysis

Statistical analysis was performed using Bell Curve for Excel version 4.00 (Social Survey Research Information Company Ltd., Tokyo, Japan) and Excel (Microsoft Corporation, Redmond, WA, USA). All data are expressed as the mean ± standard error of the mean. Exploratory behavior frequency, rotarod test, and VCM were compared between the placebo, mild TD, and severe TD groups using the Kruskal–Wallis post hoc Steel–Dwass test. Spearman's correlation coefficient was used to evaluate the correlation of VCM with exploratory behavior and the rotarod test, in haloperidol‐treated rats, not including placebo‐treated control rats. Relative ratios in Western blotting for dynorphin, enkephalin, dopamine D1 and D2 receptors, and TH with GAPDH between the three groups were compared using one‐way analysis of variance (ANOVA) and post hoc Dunnett tests. *p* < 0.05 was considered statistically significant.

## 3 Results

### 3.1 Behavioral Analysis

**FIGURE 1:**
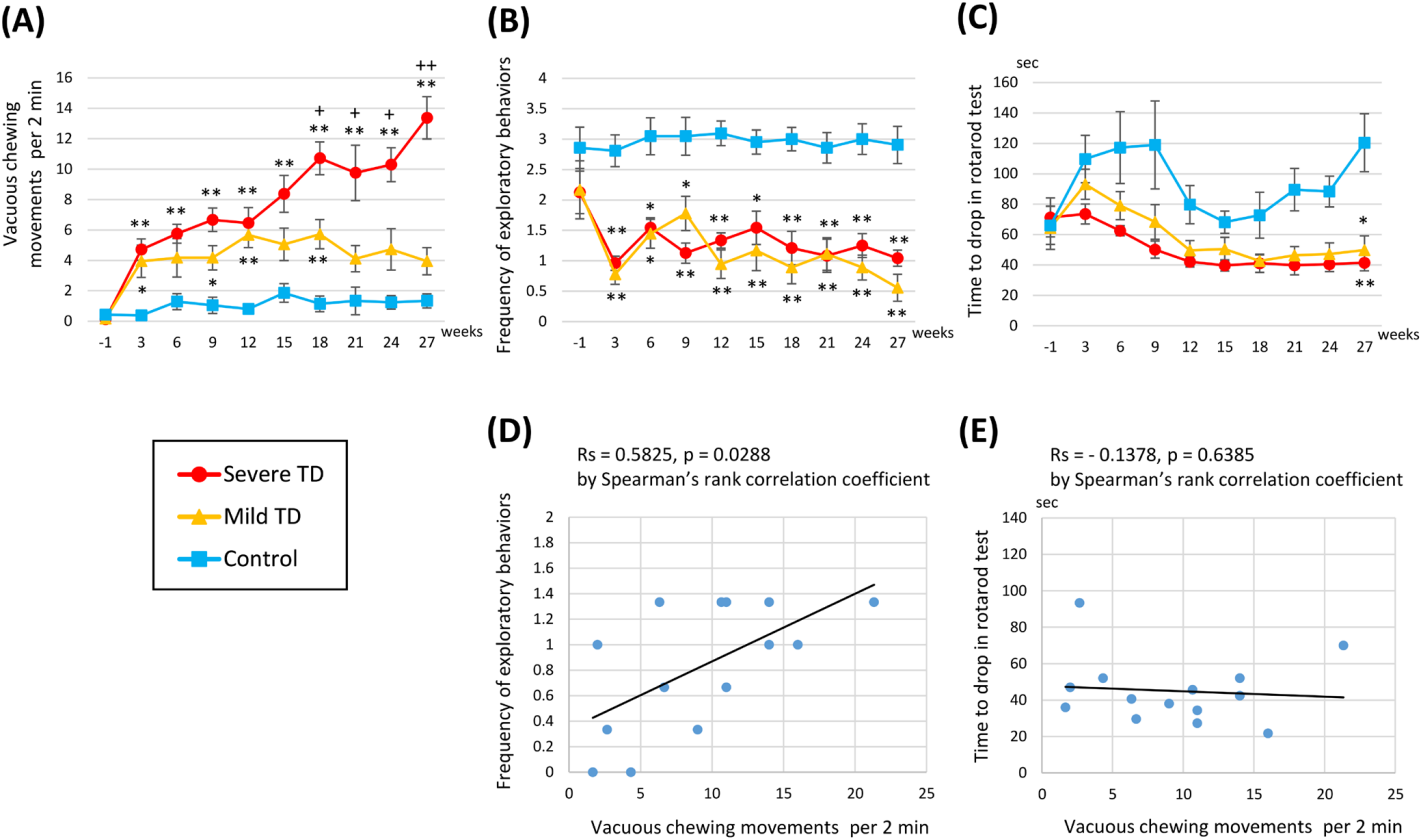
Behavioral analyses in chronic haloperidol‐treated rats. (A) Vacuous chewing movements (VCMs) per 2 min gradually increased with repeated haloperidol treatment in the severe TD group. (B) The frequency of exploratory behavior decreased after treatment initiation. (C) Performance in the rotarod test deteriorated with haloperidol treatment. Statistical analysis was performed only for week 27. (D) There was a positive correlation between the number of VCMs and the frequency of exploratory behaviors in haloperidol‐treated rats. (E) There was no association between the number of VCMs and rotarod test performance in haloperidol‐treated rats. *p < 0.05 and **p < 0.01 compared with the control group; +*p* < 0.05 and ++*p* < 0.01 compared with mild TD group via Kruskal–Wallis and post hoc Steel–Dwass tests. Error bars represent the standard error of the mean. TD, tardive dyskinesia.

All rats treated with haloperidol (*n* = 14) developed VCM, which started during the first week of treatment and gradually increased. The average VCM during the final 3 weeks was eight or more times/2 min in eight rats (severe TD) and less than eight times/2 min in six rats (mild TD; Figure 1A).

Haloperidol treatment significantly reduced the frequency of exploratory behavior compared to the control group soon after treatment initiation, indicating that haloperidol administration caused akinesia (typical DIP), although the frequency did not decrease with long‐term treatment. The decrease in exploratory behavior was not significantly different between the severe and mild TD groups (Figure 1B). However, the number of VCMs and the frequency of exploratory behavior in haloperidol‐treated rats were positively correlated (Figure 1D).

Performance in the rotarod test significantly deteriorated with haloperidol treatment compared to the control group (Figure 1C). There was no association between the number of VCMs and rotarod test performance in haloperidol‐treated rats (Figure 1E). Moreover, the time to drop in the rotarod test markedly fluctuated in control rats (Figure 1C). After several sessions, the rats jumped off to the floor or onto the large disk on the rotarod device instead of dropping. We carefully interfered with these behaviors at the end of the treatment period. None of the haloperidol‐treated rats showed such behaviors. Because of these unexpected fluctuations, we performed statistical analysis only for the results of the last rotarod test (in week 27).

### 3.2 Western Blot Analyses of Striatal Tissue

**FIGURE 2:**
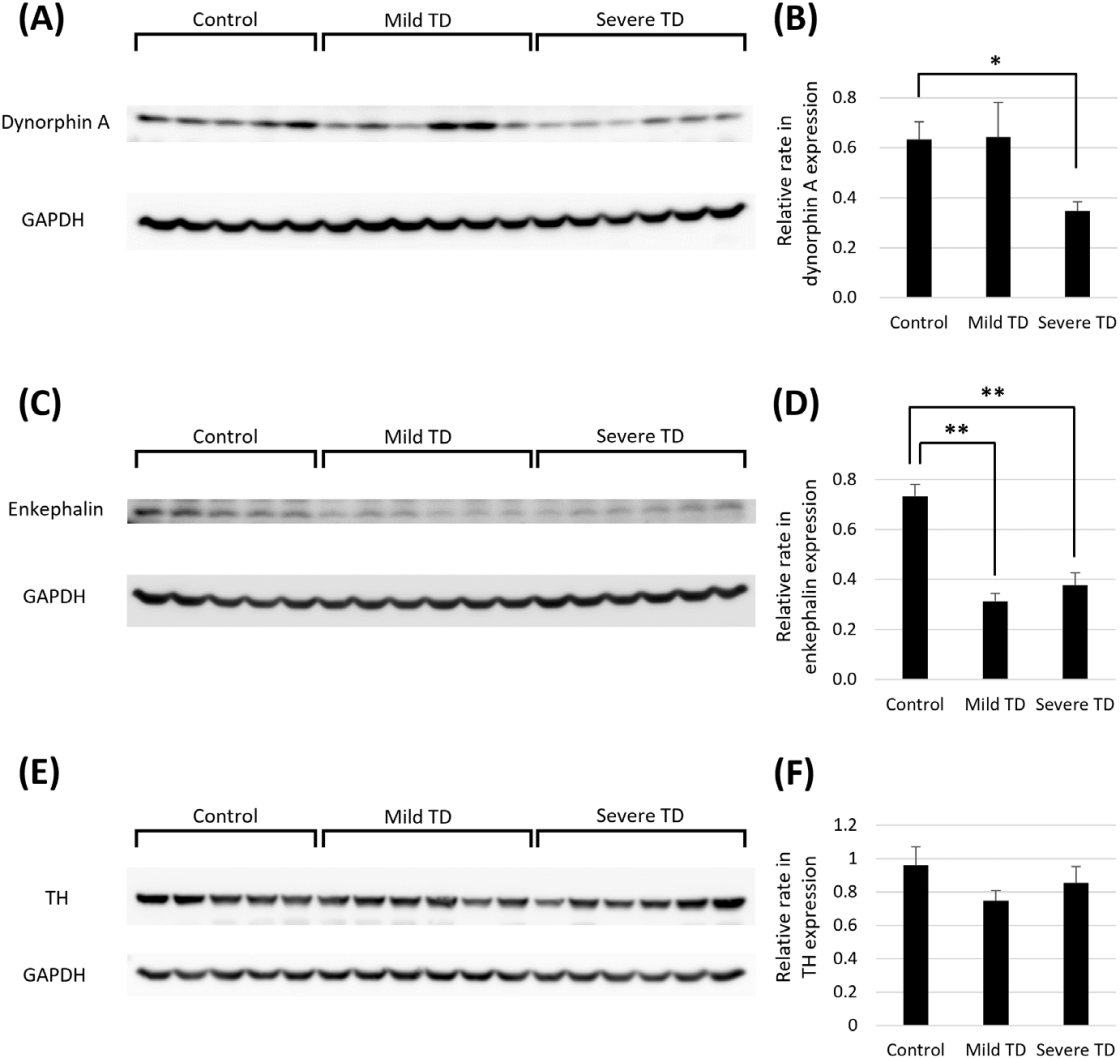
Western blot analyses of rat striatal tissue. (A, B) Dynorphin A expression was decreased in the severe TD group. (C, D) Enkephalin expression was decreased in the mild and severe TD groups. (E, F) TH expression was not significantly different among groups. *p < 0.05; **p < 0.01 via one‐way ANOVA and post hoc Dunnett tests. Error bars represent the standard error of the mean. GAPDH, glyceraldehyde 3‐phosphate dehydrogenase; TD, tardive dyskinesia; TH, tyrosine hydroxylase.

**FIGURE 3:**
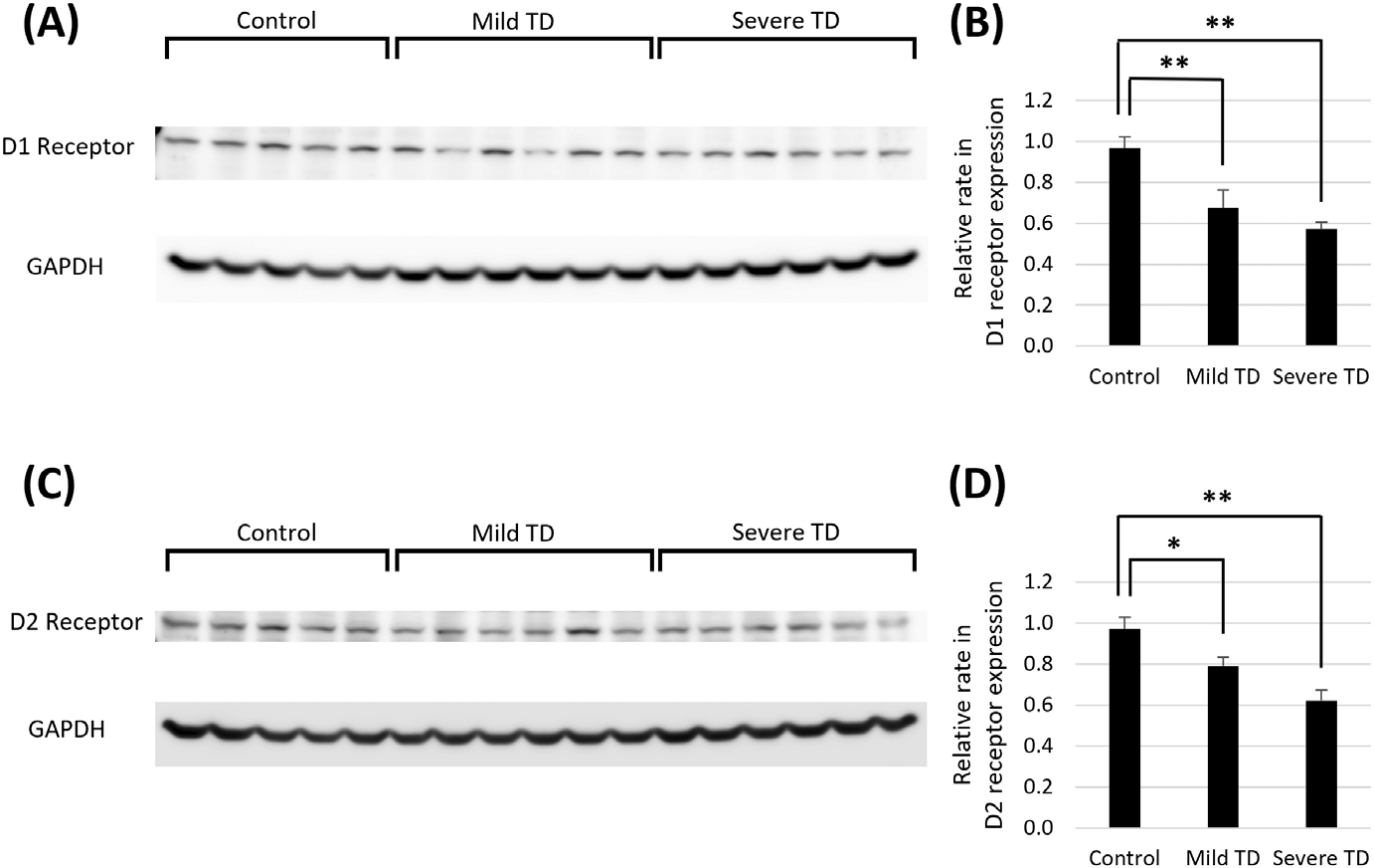
Western blot analyses of rat striatal tissue. (A, B) Dopamine D1 receptor expression was decreased in the mild and severe TD groups. (C, D) Dopamine D2 receptor expression was also decreased in the mild and severe TD groups. *p < 0.05; **p < 0.01 via one‐way ANOVA and post hoc Dunnett tests. Error bars represent the standard error of the mean. GAPDH, glyceraldehyde 3‐phosphate dehydrogenase; TD, tardive dyskinesia.

Dynorphin A expression was significantly decreased in the severe TD group compared to the control group (Figure 2A,B, Figures S1A and S2A). In contrast, enkephalin expression was significantly decreased in both the mild and severe TD groups compared to the control group (Figure 2C,D, Figures S1B and S2B). TH expression did not significantly differ among the three groups (Figure 2E,F, Figures S1C and S2C). The expressions of dopamine D1 and D2 receptors were significantly decreased in both the mild and severe TD groups compared to the control group (Figure 3A–D, Figures S1D,E and S2B,D).

## 4 Discussion

In this study, all haloperidol‐treated rats exhibited VCM and displayed decreased exploratory behavior and poor performance in the rotarod test. Dynorphin and enkephalin expression was differentially altered according to TD severity, suggesting that both direct and indirect pathways are involved in haloperidol‐induced movement disorders.

VCM appeared after haloperidol administration and gradually increased in the severe TD group. Since clinical TD also becomes apparent after chronic administration of antipsychotics [16], VCM in rats may be comparable to TD in humans. In contrast, exploratory behavior decreased soon after haloperidol administration and remained at approximately the same level during the experimental period. Since clinical DIP appears early after antipsychotic administration [2], decreased exploratory behavior in rats may be equivalent to DIP in patients. Therefore, we established a rat model presenting both DIP and TD.

The number of VCMs was positively correlated with the degree of exploratory behavior in haloperidol‐treated rats, indicating that rats with milder DIP exhibited more severe TD. This matches well with the previous hypothesis that D2 receptor blockade increases the excitability of indirect pathway neurons, resulting in DIP, and that compensatory D2 receptor hypersensitivity inactivates indirect pathway striatal neurons, resulting in TD [7].

Fluctuations in the time to drop in the rotarod test for control rats may have been due to the following reason. As the rats were newly introduced to rotarod testing, the time to drop was long. After several sessions, the rats got accustomed to the test and left the rotor via other means than dropping, and the time to drop shortened. We carefully interfered with this behavior, after which the time to drop recovered to the normal level. Interestingly, haloperidol‐treated rats did not show such cunning behavior. Learning ability might be impaired by haloperidol treatment, but this was not evaluated in the present study and should be examined in the future.

Haloperidol treatment decreased dynorphin expression in the striatum in the severe TD group. This indicates that alteration of direct pathway activity may be related to TD severity rather than DIP. In contrast, enkephalin expression in the striatum decreased both in the mild and severe TD groups, suggesting its relation to DIP. The non‐significant difference in TH expression among the three groups suggested that there was no dopaminergic nerve impairment. These striatal protein expression results suggest that both direct and indirect pathways are involved in haloperidol‐induced motor symptoms. Although previous reports on DIP and TD hypothesized that altered indirect pathway activity underlies these movement disorders [5, 17, 18, 19], the present findings suggest that the direct pathway plays a significant role as well. Recently, Abe et al. [20] demonstrated that GABAergic nerve terminals in the external segment of the globus pallidus and substantia nigra pars reticulata were enlarged, and vesicular GABA transporter was overexpressed in the striatum of haloperidol‐treated mice. Their study also suggested the involvement of both direct and indirect pathways in haloperidol‐induced movement disorders.

Furthermore, expression of dopamine D1 and D2 receptors decreased in both the mild and severe TD groups, which is inconsistent with the previous hypothesis that compensatory D2 receptor hypersensitivity underlies TD. Decreases in D2 receptor expression may be associated with DIP rather than TD. Alternatively, protein expression does not necessarily reflect actual neuronal sensitivity because receptor localization is important in terms of sensitivity to dopamine [21, 22].

A limitation of this study was that we investigated only protein expression, but not neuronal activity. Future studies should examine mRNA expression and action potentials using electrophysiological techniques to elucidate the exact roles of direct and indirect pathway striatal neurons in DIP and TD.

## Author Contributions

I.K. conducted experiments, analyzed data, interpreted results, and wrote the manuscript. H.N. obtained a grant for the research; conducted experiments; analyzed data; interpreted results; and wrote, reviewed, and revised the manuscript. T.N., T.K., S.S., H.H., and C.S. conducted experiments and reviewed the manuscript. M.T. obtained a grant for this research, designed the study, and reviewed the manuscript.

## Ethics Statement

Approval of the Research Protocol by an Institutional Reviewer Board: This animal study was reviewed and approved by the Hirosaki University Graduate School of Medicine, Japan. Animal Studies: Experimental procedures complied with the Principles of Laboratory Animal Care (NIH Publication Vol 25, No. 28 revised 1996; https://grants.nih.gov/grants/guide/notice‐files/not96‐208.html) and guidelines for animal research issued by the Physiological Society of Japan and Hirosaki University Graduate School of Medicine.

## Consent

The authors have nothing to report.

## Conflicts of Interest

Haruo Nishijima received a research grant from Sumitomo Pharma Co. Ltd. The other authors declare that they have no competing financial interests or personal relationships that could have influenced the work reported in this study.

## Supporting information


**Figure S1.** Entire membranes with size markers for Western blotting experiments using antibodies for dynorphin A (A), enkephalin (B), TH (C), dopamine D1 receptor (D), and dopamine D2 receptor (E). Numbers indicate the molecular weight (kDa). Red arrowheads indicate the targeted bands for each antibody. TH, tyrosine hydroxylase.

**Figure S2.** Entire membranes with size markers for Western blotting experiments evaluating GAPDH expression with samples for dynorphin A (A), enkephalin and dopamine D2 receptor (B), TH (C), and dopamine D1 receptor (D). Numbers indicate the molecular weight (kDa). Red arrowheads indicate the targeted bands. In A, B, and D, GAPDH expression and size markers are shown for the same membrane exposed for different times. Lines are placed to distinguish the two different time figures. GAPDH, glyceraldehyde 3‐phosphate dehydrogenase. TH, tyrosine hydroxylase.

Data S1. Raw data of behavioral analyses

Data S2. Raw data of Western blot analyses

## Data Availability

The data that support the present results, including pictures of the entire membranes with size markers for Western blotting experiments, are available in the [Link], [Link] of this article.
