# Supplementary figures and images for: Co‐Expression of Tardive Dyskinesia and Drug‐Induced Parkinsonism in Rats Chronically Treated With Haloperidol
Source: Neuropsychopharmacol Rep. 2025 Jan 9;45(1):e12524. doi: 10.1002/npr2.12524 (PMC11717661; doi:10.1002/npr2.12524)

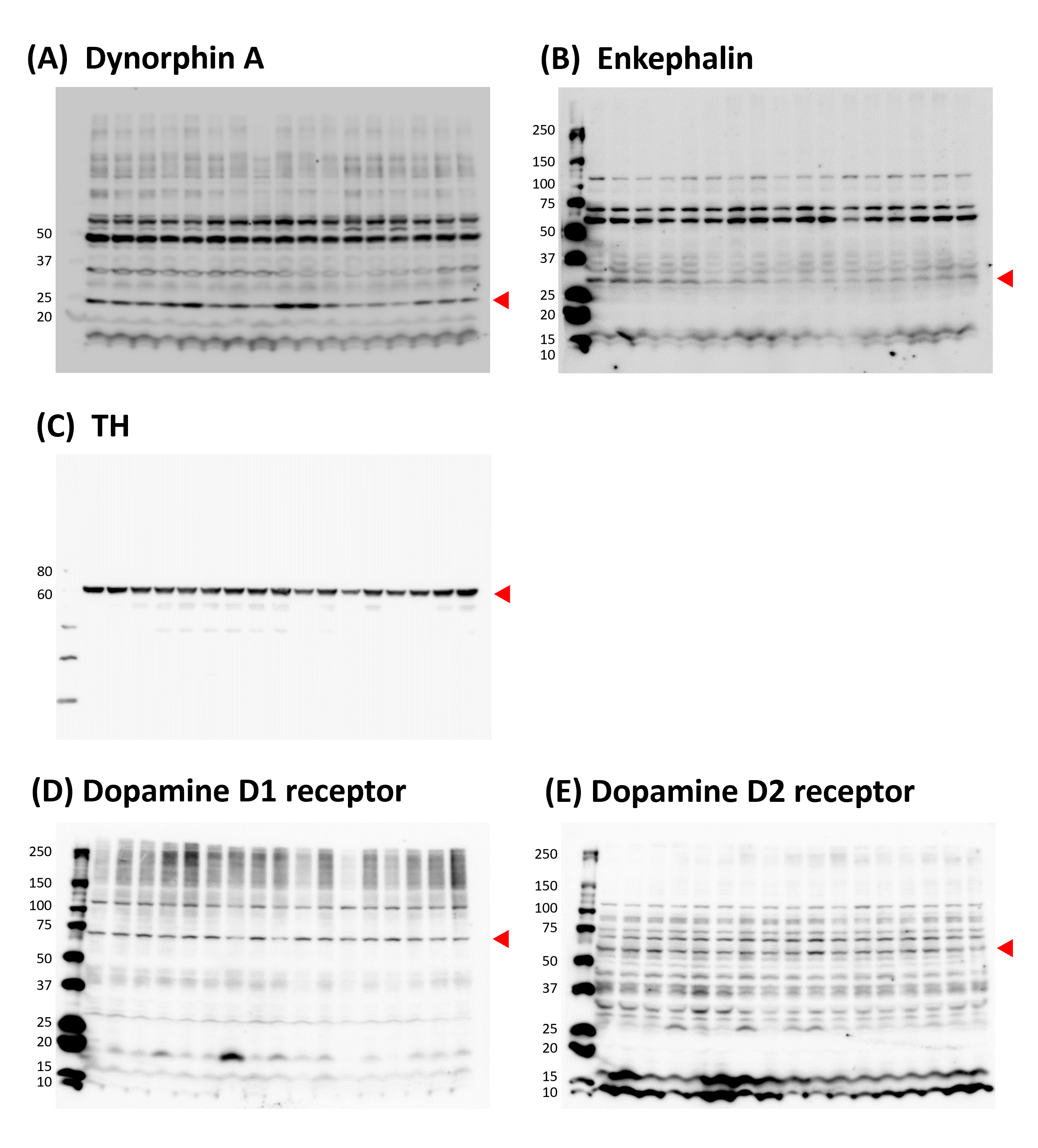

Supplement: Supplementary file 1 — Figure S1. Entire membranes with size markers for Western blotting experiments using antibodies for dynorphin A (A), enkephalin (B), TH (C), dopamine D1 receptor (D), and dopamine D2 receptor (E). Numbers indicate the molecular weight (kDa). Red arrowheads indicate the targeted bands for each antibody. TH, tyrosine hydroxylase. [file NPR2-45-e12524-s001.tif]

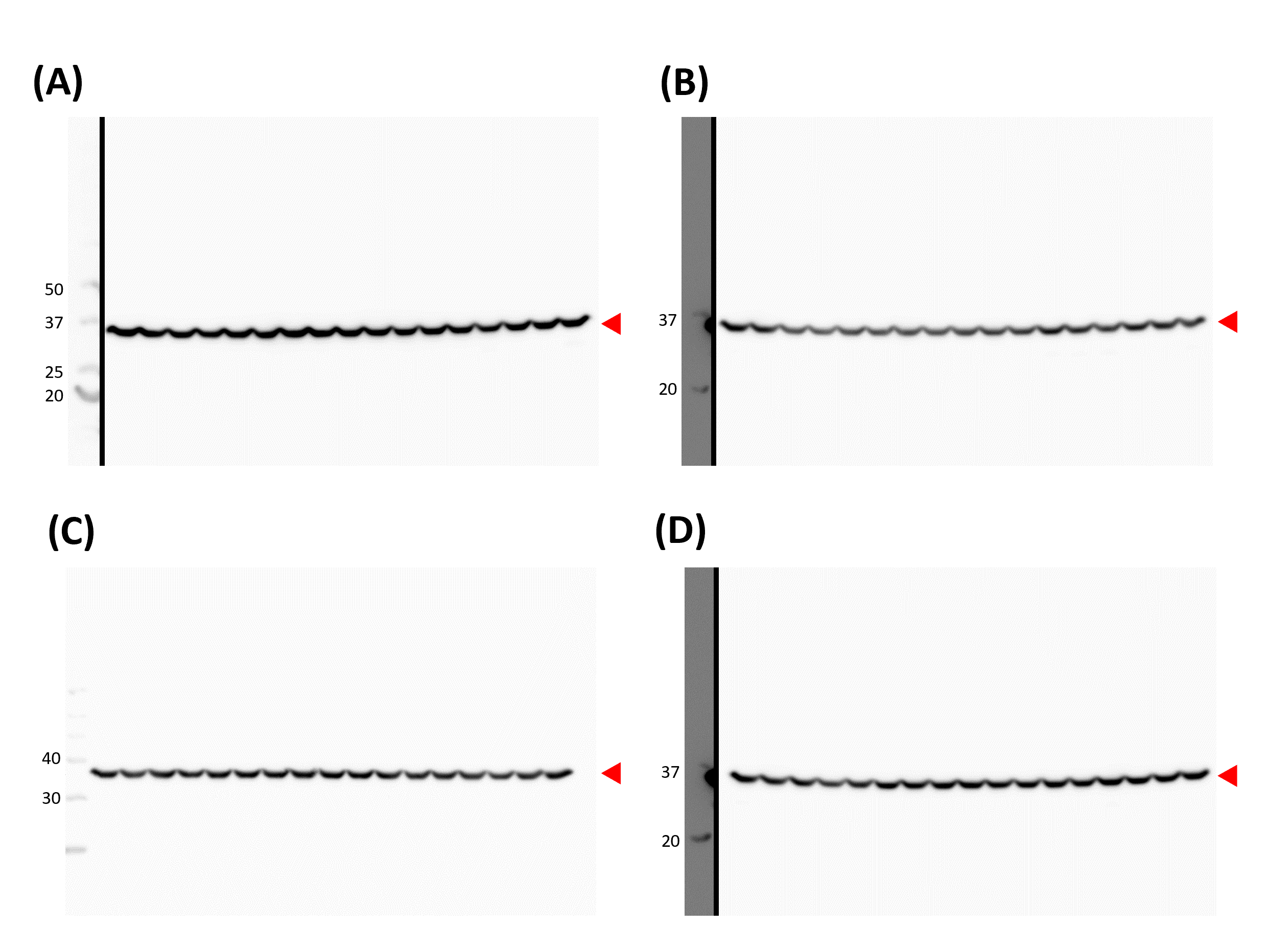

Supplement: Supplementary file 2 — Figure S2. Entire membranes with size markers for Western blotting experiments evaluating GAPDH expression with samples for dynorphin A (A), enkephalin and dopamine D2 receptor (B), TH (C), and dopamine D1 receptor (D). Numbers indicate the molecular weight (kDa). Red arrowheads indicate the targeted bands. In A, B, and D, GAPDH expression and size markers are shown for the same membrane exposed for different times. Lines are placed to distinguish the two different time figures. GAPDH, glyceraldehyde 3‐phosphate dehydrogenase. TH, tyrosine hydroxylase. [file NPR2-45-e12524-s003.tif]
